# Synaptogenesis and outer segment formation are perturbed in the neural retina of Crx mutant mice

**DOI:** 10.1186/1471-2202-6-5

**Published:** 2005-01-27

**Authors:** Eric M Morrow, Takahisa Furukawa, Elio Raviola, Constance L Cepko

**Affiliations:** 1 Department of Genetics and Howard Hughes Medical Institute, Harvard Medical School, New Research Building, Room 360K, NRB, Room 360K, 77 Avenue Louis Pasteur, Boston, Massachusetts, 02115, USA; 2 Department of Neurobiology, Harvard Medical School, 220 Longwood Avenue, B2-201, Boston, Massachusetts, 02115, USA; 3 The 4th Department, Osaka Bioscience Institute, 6-2-4 Furuedai, Suita, Osaka, Japan

## Abstract

**Background:**

In Leber's congenital amaurosis (LCA), affected individuals are blind, or nearly so, from birth. This early onset suggests abnormal development of the neural retina. Mutations in genes that affect the development and/or function of photoreceptor cells have been found to be responsible in some families. These examples include mutations in the photoreceptor transcription factor, Crx.

**Results:**

A Crx mutant strain of mice was created to serve as a model for LCA and to provide more insight into Crx's function. In this study, an ultrastructural analysis of the developing retina in Crx mutant mice was performed. Outer segment morphogenesis was found to be blocked at the elongation stage, leading to a failure in production of the phototransduction apparatus. Further, Crx-/- photoreceptors demonstrated severely abnormal synaptic endings in the outer plexiform layer.

**Conclusions:**

This is the first report of a synaptogenesis defect in an animal model for LCA. These data confirm the essential role this gene plays in multiple aspects of photoreceptor development and extend our understanding of the basic pathology of LCA.

## Background

Photoreceptor cells play a primary role in vision by capturing light energy and converting it into neural stimuli. These sensory neurons are a shared element in all organisms capable of sensing light. In humans, genetic diseases of the eye are common and the primary site of disease is most frequently photoreceptors (for review see [1-3]).

Photoreceptors have been well studied, particularly with respect to the biochemistry and physiology of phototransduction. Insight into the development of vertebrate photoreceptors, however, has lagged behind our understanding of function. Only recently have the first molecular mechanisms regulating photoreceptor development been identified (for review see, [2,4]). Crx (cone-rod homeobox) is an otx-family homeobox gene expressed predominantly in photoreceptors, from early in their development through to the adult ages [5-7]. Crx gene expression is critically dependent upon Otx2, another member of the same homeobox family which is expressed in early photoreceptor cells [8]. In rod photoreceptors, Crx appears to work in concert with Nrl, a leucine zipper protein that is also restricted in its expression in the retina to rod photoreceptors [9]. Many photoreceptor-specific genes have putative Crx-binding elements in their regulatory regions [10], including rhodopsin [11] and arrestin [12]. Mutations in Crx have been associated with several human diseases that lead to blindness, including cone-rod dystrophy 2 [6,13,14], retinitis pigmentosa [14], and LCA [14-16]. Based on these data, Crx was hypothesized to play a critical role in the differentiation and maintainance of photoreceptor cells [5,7].

LCA is the most severe genetic disease of photoreceptors (see [17], for recent review). Affected infants exhibit a complete or near complete absence of vision from birth. Mutations in retinal specific genes, such as Crx, have been associated with LCA [14,15], as well as GUCY2D [18], RPE65 [19], AIPL-1 [20], CRB-1 [21], and RPGRIP-1 [22]. There also may be as many as three additional genetically linked loci where genes have not been identified [23]. Crx mutations in LCA are varied, and include a putative dominant mutation that is proposed to encode a dominant-negative form of Crx [14,15]. Recessive mutations also have been reported and at least one allele encodes a protein with decreased DNA-binding activity [16]. Histopathological and ultrastructural studies of LCA should enable a better understanding of the disease process, and the design of suitable therapies. Few such studies exist for human LCA (reviewed in [17]) and the majority of such studies examine the globes of adults with LCA, after the tissue has undergone secondary changes. Only a single study exists where the developing eye of an infant was examined [24]. Animal models for LCA have recently been reported and have already served to broaden our understanding of the pathology of this disease [25-28]. Since LCA is a clinically and genetically heterogeneous disorder, additional mouse models are in order to allow a full understanding of the many ways in which photoreceptor development can go awry.

In addition to their importance as a locus of disease, photoreceptor cells serve as an excellent model for studies in neuronal differentiation. Photoreceptor cells are highly polarized. At their apex, these neurons have a membranous outer segment, which contains proteins involved in the phototransduction cascade. Loss of function mutations in rhodopsin [29], or the structural protein, peripherin [30], result in an inability to form outer segments. At the other extremity, photoreceptors terminate with synaptic endings that make contact with the processes of horizontal and bipolar cells [31,32]. Rod spherules establish an invaginating synapse with rod bipolar dendrites and axonal endings of horizontal cells. This synapse is characterized by the presence of a ribbon in the presynaptic cytoplasm. Cone pedicles make invaginating synapses with the dendrites of on-cone bipolar cells and horizontal cells and basal junctions with the dendrites of off-cone bipolar cells. The factors regulating the formation of the photoreceptor synapses are completely unknown. At least one photoreceptor synaptic protein, HRG4, contains a potential Crx target sequence in its transcriptional regulatory sequence [33].

Few studies of LCA animal models have extended their examination of retinal pathology to the ultrastructural level. Certain features of neuronal differentiation, such as synapse formation, can be detected definitively at this level of analysis. With the hope of understanding the neuropathology of LCA in greater detail, we have analyzed the differentiation of the outer retina in Crx-/- mice at the ultrastructural level. These retinas exhibit several prominent defects. Crx-/- photoreceptors demonstrate a complete block in outer segment formation at the elongation stage. Further, these cells exhibit abnormal synaptic morphology, thereby broadening the function of Crx to photoreceptor synaptogenesis. This represents the first report strongly implicating the process of synapse formation in LCA.

## Results

### Multiple pathologies in the outer segment layer in Crx-/- mice

**Figure 1:**
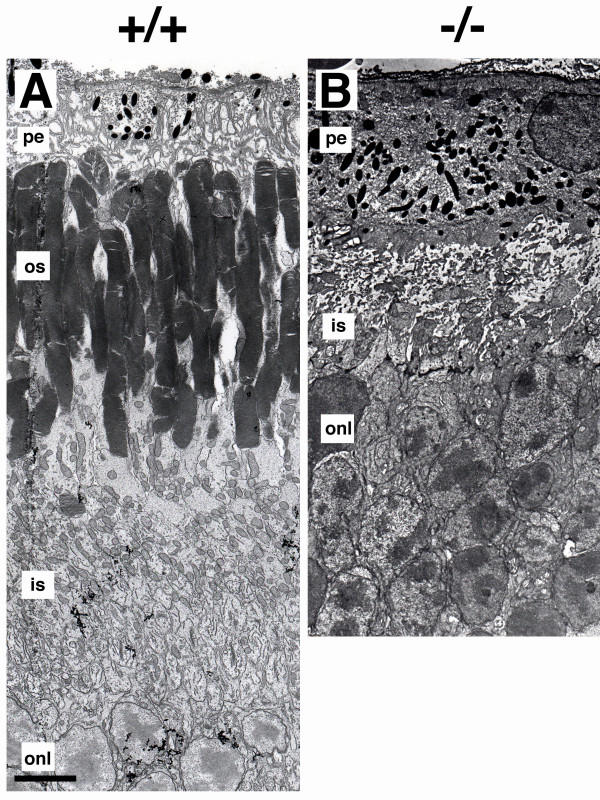
Transmission electron microscopy of the outer retina at P21 in (A) Crx+/+ and (B) Crx-/- retinas. pe, pigmented epithelium. os, outer segments. is, inner segments. onl, outer nuclear layer with photoreceptor nuclei. Scale bar = 5 μm for A and B.

**Figure 2:**
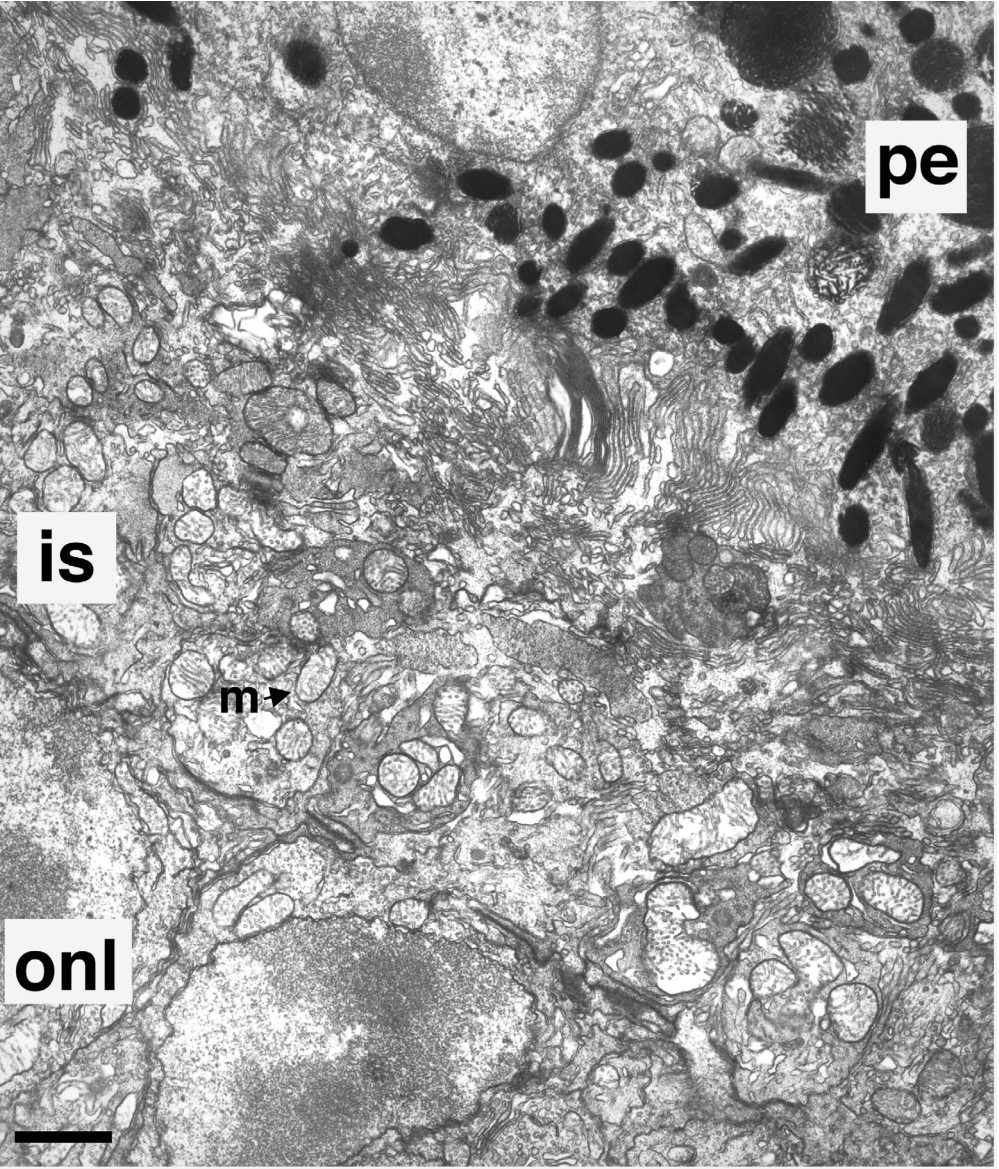
Transmission electron micrograph of the outer segment layer of Crx-/- retina at P21. Inner segments of Crx-/- photoreceptors exhibit numerous mitochondria (m indicated by arrow) as in Crx+/+ (Figure 1A). pe, pigmented epithelium. is, inner segments. onl, outer nuclear layer. Scale bar = 2 μm.

A standard knock-out protocol was used to generate mice in which the homeodomain of Crx-/- was targeted and deleted. Generation of these Crx-/- mice has been reported elsewhere [34]. In this study, in order to characterize further the role of Crx in photoreceptor morphogenesis, the outer retinae from Crx-/- mice were examined using transmission electron microscopy. At postnatal day 21 (P21), when Crx+/+ photoreceptors exhibited robust outer segments (Figure 1A, os), Crx-/- retinas were without a recognizable outer segment layer (Figure 1B). Crx-/- photoreceptors had inner segments, demonstrating at least limited photoreceptor polarization in the Crx mutant, but the inner segments were extremely short (Figure 2). Furthermore, the majority of inner segments showed some morphological differentiation, having approximately as many mitochondria as the control (Figure 1 and 2). Occasionally, an inner segment undergoing lysis was noted, appearing swollen or with vacuoles and swollen mitochondria (data not shown).

**Figure 3:**
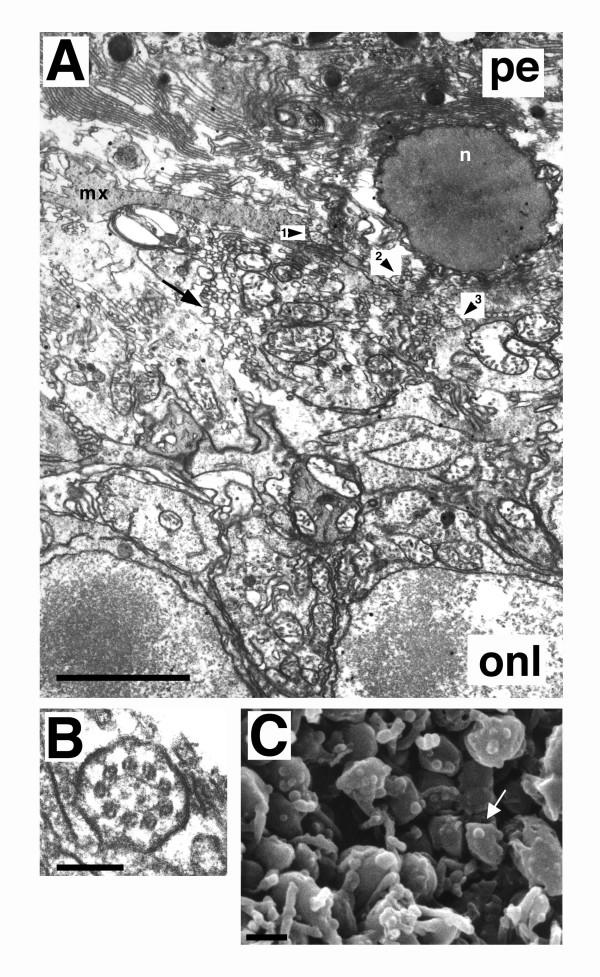
Transmission electron micrograph of Crx-/- retina at P21 (A and B), and scanning electron micrograph of Crx-/- at P10 (C) of outer segment layer. (A) Evidence of ciliogenesis in the photoreceptor layer of Crx-/- retina. Nonmotile connecting cilia were observed in cross section (arrowheads 1,2, and 3, for examples). Connecting cilium 1 (arrowhead 1) demonstrated seven microtuble doublets, while cilium 2 and cilium 3 exhibited nine. In A, a displaced cell nucleus (n) appearing pyknotic and abnormal deposition of matrix (mx) material of unknown identity were seen, along with large amounts of membranous vesicles (arrow) which filled the photoreceptor space and appeared to be released from inner segments. Scale bar = 3.7 μm. (B) Nonmotile connecting cilium in cross section, from a Crx-/- photoreceptor, demonstrating characteristic 9+0 radial array of microtubule doublets. Scale bar = 88 nm. (C) Scanning electron micrograph (SEM) of membranous vesicles (arrow shows one example) shed from inner segments of Crx-/- photoreceptors at P10. Figure shows inner segments viewed from the scleral side with the pigmented epithelium removed. Scale bar = 1 μm.

Photoreceptor inner segments and outer segments are joined by a non-motile connecting cilium that exhibits a characteristic 9 + 0 arrangement of microtubule doublets when viewed in cross-section. At P21, in Crx-/- retinas, numerous cross sections of connecting cilia were noted (Figure 3A and 3B). Sporadically, connecting cilia contained other than the typical complement of microtubule doublets. For example, in Figure 3A, the connecting cilium labelled by arrowhead 1, shows 7 + 0 doublets. The majority exhibited the characteristic 9 + 0 doublets (arrowhead 2 and 3 in Figure 3A and Figure 3B). These observations indicate that in addition to inner segment formation, ciliogenesis is also largely intact in Crx-/- photoreceptors. Further, in Crx-/- retinas the retinal pigmented epithelium (PE) appeared normal, at least up to P21 (data not shown), the oldest age examined.

In addition to the complete absence of outer segments, Crx-/- retinas exhibited three other notable pathologies in the outer segment layer. First, an abnormal deposition of matrix of unknown identity was noted (Figure 3A, mx). Second, sporadically displaced nuclei were found residing in the space abutting the PE. Occasionally, these nuclei appeared pyknotic (Figure 3A, n); but, more frequently exhibited the heterochromatin pattern typical of photoreceptors (data not shown), strongly suggesting that they belonged to ectopic photoreceptors. The third pathological entity noted in the outer segment layer were numerous small vesicles (Figure 3A arrow) 100 to 200 nm in diameter. They appeared to be emerging from the inner segments, as scanning electron microscopic images showed spherical structures budding from the inner segments (Figure 3C, arrow).

**Figure 4:**
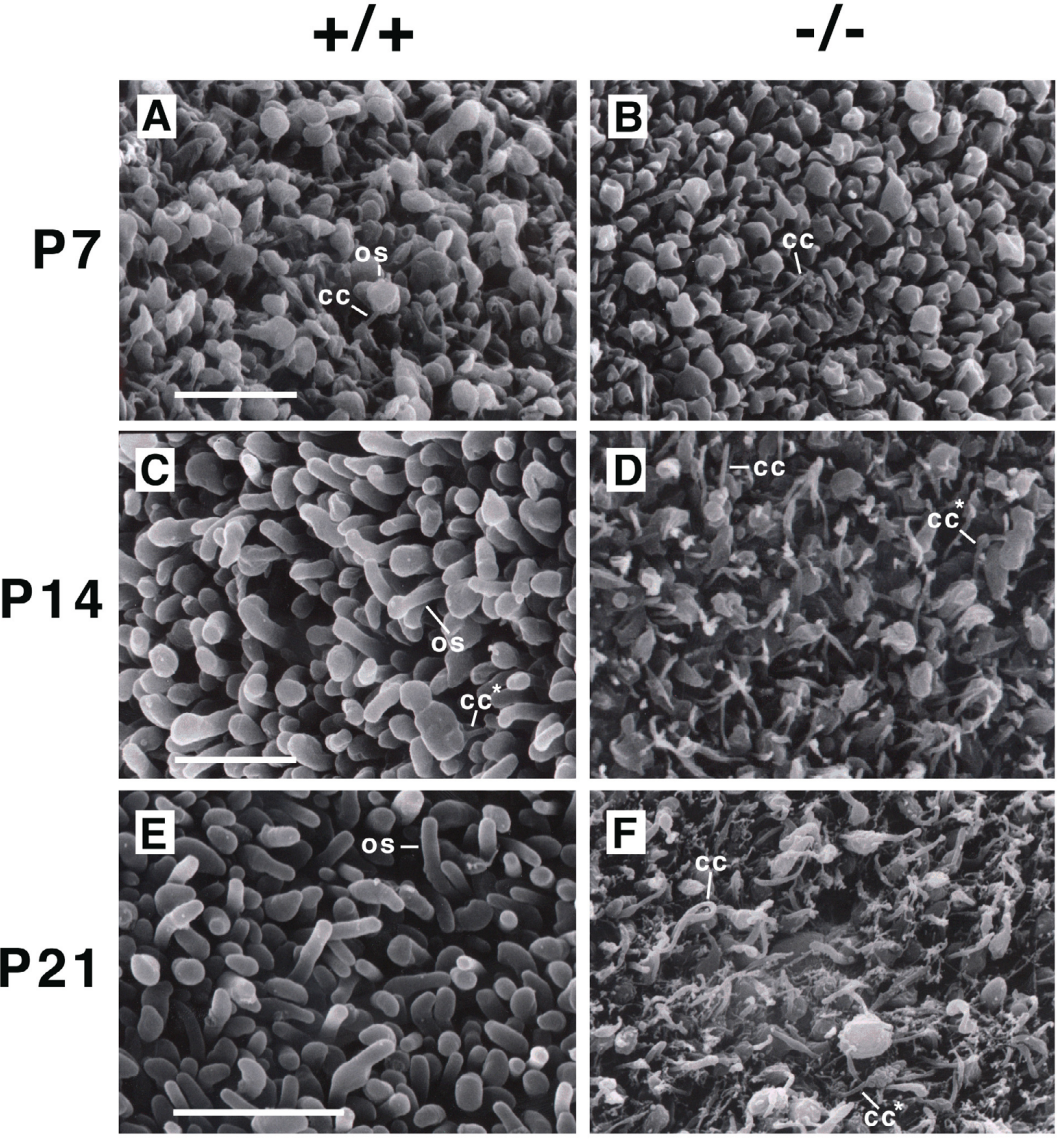
Outer segment morphogenesis in Crx-/- photoreceptors. Scanning electron microscopy of developing photoreceptors viewed from the scleral side with the pigmented epithelium removed at P7, P14, and P21 for Crx+/+ (A, C, and E) and Crx-/- (B, D, F) littermates. In Crx+/+ retina, numerous connecting cilia (A, cc) were evident at P7 with rudimentary outer segments. After P7, in Crx+/+ outer segment elongation occurs. Initially, outer segments have a paddle-like structure (C, os) which is later shed (E, os). In Crx-/- photoreceptors, few connecting cilia were observed at P7 (B, cc). After P7, connecting cilia were more numerous and occasionally a malformed outer segment was noted extending from a connecting cilium (D and F, cc*). These were rarely observed in Crx+/+ and only at P14 (C, cc*). At P21, abnormally long connecting cilia are noted in Crx-/- (F, cc). Scale bars = 10 μm

**Figure 5:**
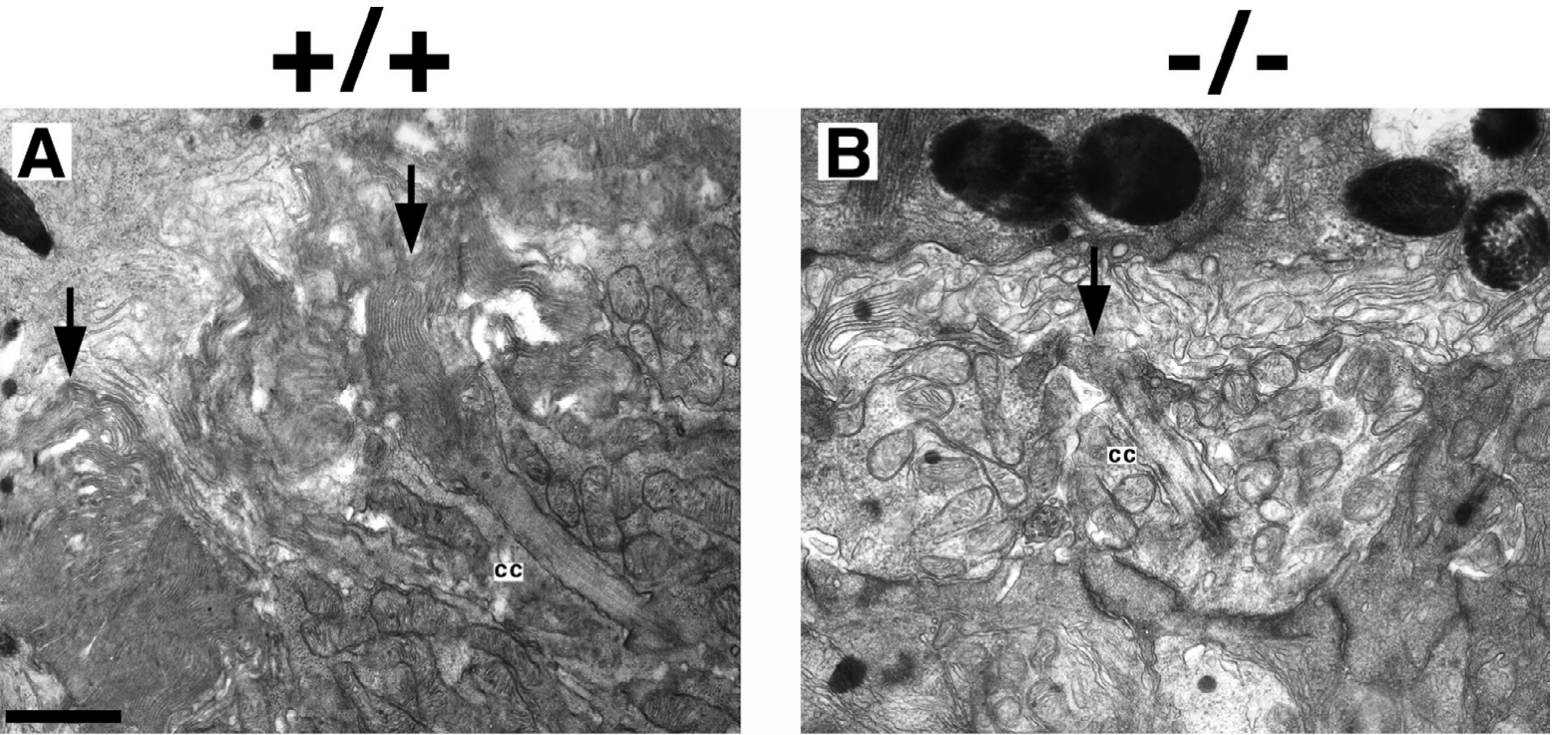
Transmission electron micrographs of Crx-/- photoreceptors in the photoreceptor layer at P7. (A) Photoreceptor layer of Crx+/+ retina demonstrating nascent outer segment structures (arrow) emerging from photoreceptor connecting cilia (cc). (B) Crx-/- photoreceptors exhibited connecting cilia (cc) at this early stage, however, nascent outer segment structures were not observed. Scale Bar = 1 μm.

In order to characterize further the morphogenesis of Crx-/- photoreceptors, the developing outer segment layer was viewed by scanning electron microscopy at P7, P14 and P21 (Figure 4). In Crx+/+ retinas, photoreceptor inner segments, connecting cilia, and the first rudimentary outer segment structures were noted at P7. In the Crx-/- retina, only an occassional connecting cilium was noted emerging from inner segments at this stage (Figure 4A and 4B). This observation was confirmed by comparison with transmission electron micrographs (Figure 5). These differences are the earliest noted differences between Crx+/+ and Crx-/- photoreceptors. At P14, elongating outer segments were noted on Crx+/+ photoreceptors, occasionally demonstrating a paddle-like structure at their apex (Figure 4C, os). In Crx-/- retinae, the vast majority of photoreceptors at this stage demonstrated connecting cilia without outer segments (Figure 4D, cc). Sporadically, Crx-/- photoreceptors would exhibit an irregular structure extending from a connecting cilium (Figure 4D, cc*) perhaps representing a malformed outer segment. Such structures were also observed at P21 (Figure 4F, cc*). These putative, abnormal outer segments were only rarely noted in Crx+/+ at P14, and never at P21 (Figure 4C and 4E, cc*). Further, in Crx-/- photoreceptors, unusually long connecting cilia were noted (Figure 4F, cc). Serial examination of Crx-/- photoreceptors at P7, P10, P14, and P21 by TEM, demonstrated a distinctive lack of any structure interpretable as orderly stacks of discs or forming discs. These data demonstrate a complete absence of normal outer segment formation in the Crx mutant mouse, and the arrest of development of the photoreceptor appendage at the elongation stage of outer segment formation.

**Figure 6:**
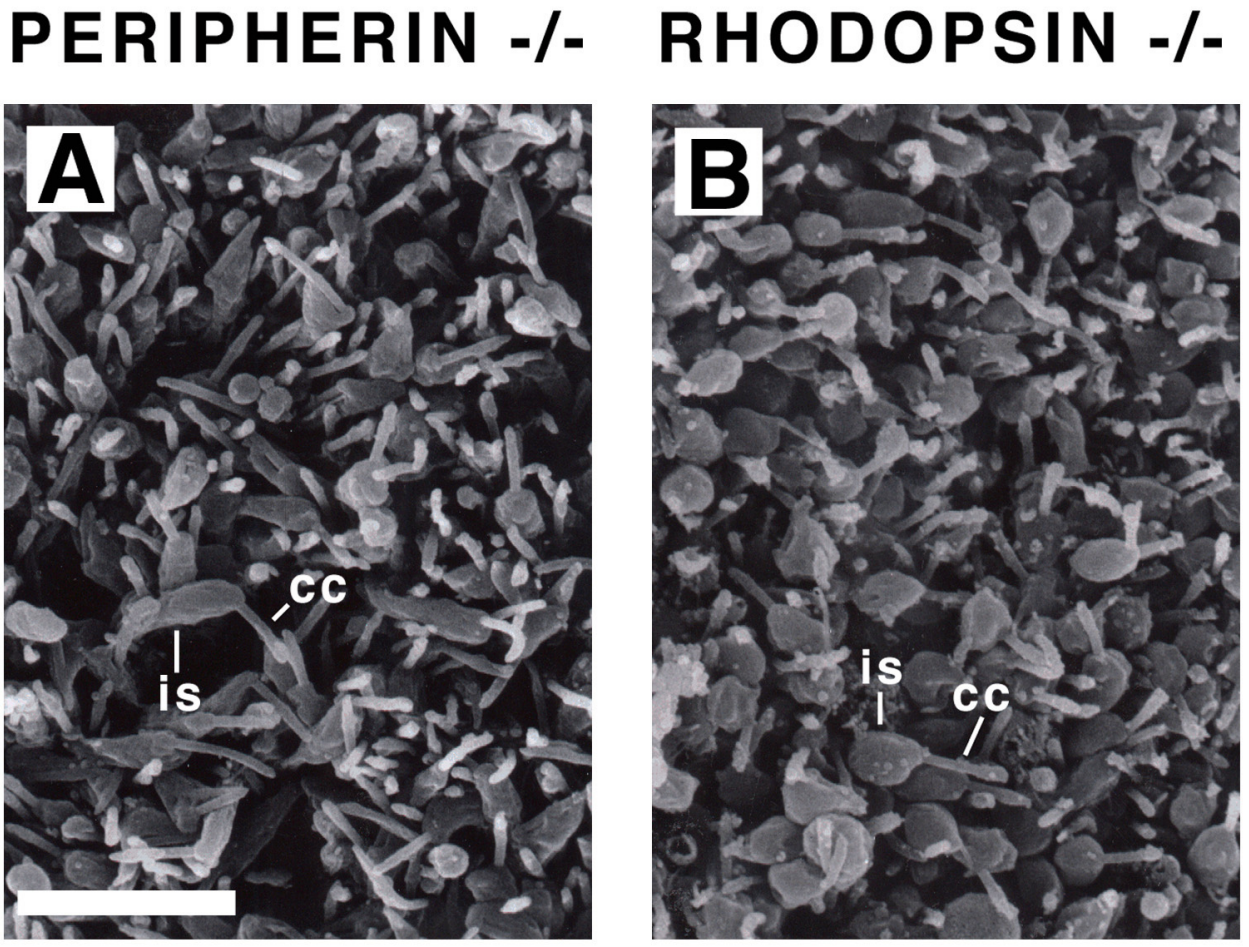
Scanning electron microscopy of peripherin-/- (A) and rhodopsin-/- (B) photoreceptors at P21, viewed from the scleral side with the pigmented epithelium removed. cc, connecting cilium. is, inner segment. Scale bar = 10 μm.

Finally, the morphology of the malformed Crx-/- photoreceptors was compared to rhodopsin-/- and peripherin-/- photoreceptors. Rhodopsin and peripherin are two photoreceptor-specific genes whose expression is significantly downregulated in the Crx-/- retinae [10,34,35]. Loss of function mutations in each of these genes separately have been reported to result in a failure to elaborate outer segments [29,30]. Photoreceptors from these two mutant mice examined by SEM from the scleral side appeared highly similar to Crx-/- photoreceptors (compare Figure 4F to Figure 6A and 6B).

### Crx is necessary for the formation of photoreceptor terminals

**Figure 7:**
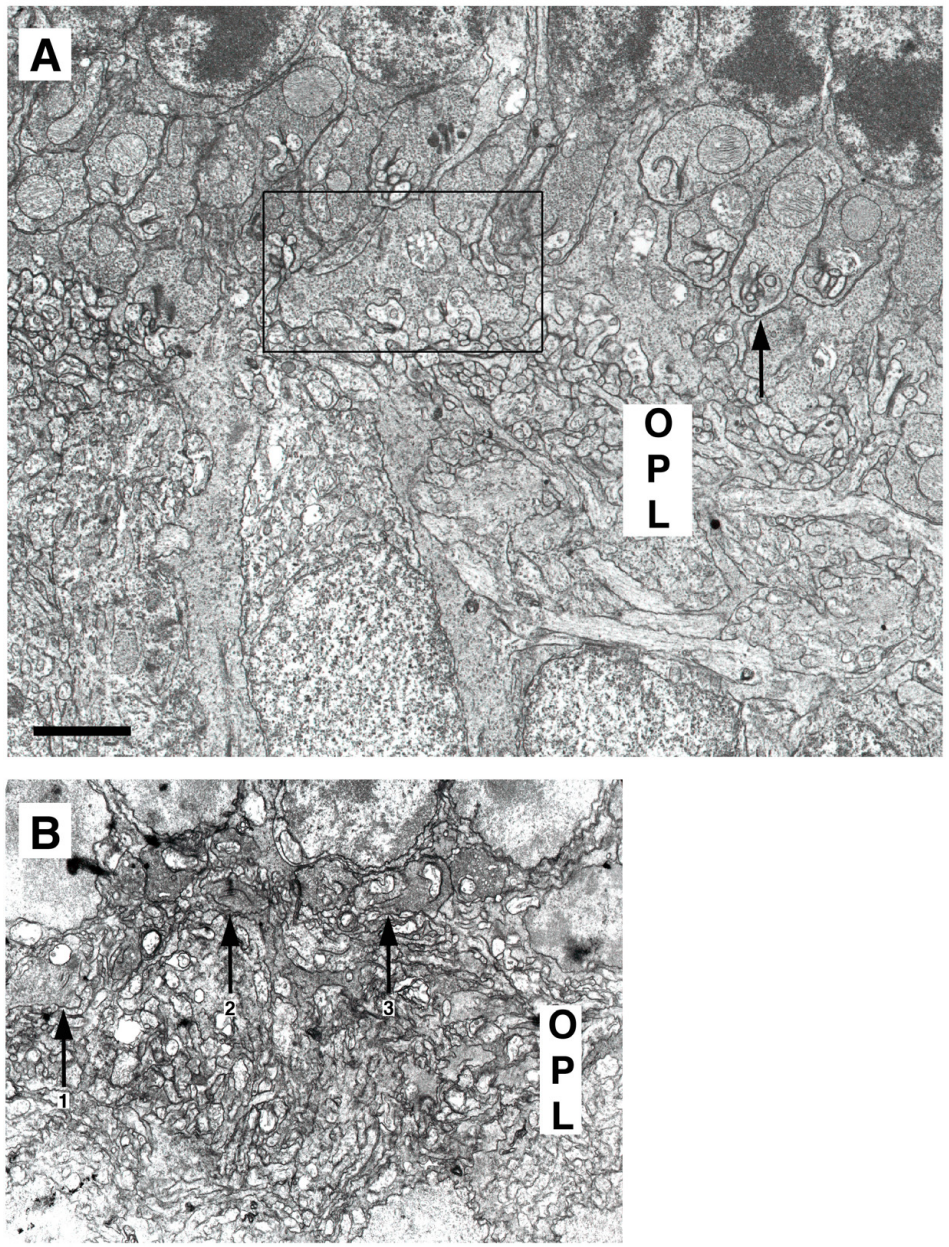
Transmission electron micrographs of the outer plexiform layer in Crx-/- retinas. (A) In Crx+/+ retina at P21, newly formed rod spherules were abundant (arrow demonstrates one example). The spherules were club-shaped and contained a single invaginating synapse with one ribbon complex. Cone terminals form large, flat pedicles that contain many invaginating synapses with separate ribbon structures. These terminals were more scarce, but apparent in Crx+/+ retinas at P21 (one example in box). (B) In the outer plexiform layer (OPL) of Crx-/- retinas, photoreceptor terminals appeared highly disorganized at P21 (arrows). Well-formed pedicles and spherules were not evident. Putative terminals containing ribbon-like structures were apparent, suggesting at least limited generation of synapse components. Many terminals appeared to contain multiple ribbon-like structures, instead of a singule ribbon. For example, terminal 1 and 2 contained two ribbons each, whereas terminal 3 appeared to contain only one. opl, outer plexiform layer. Scale bar = 2 μm.

**Figure 8:**
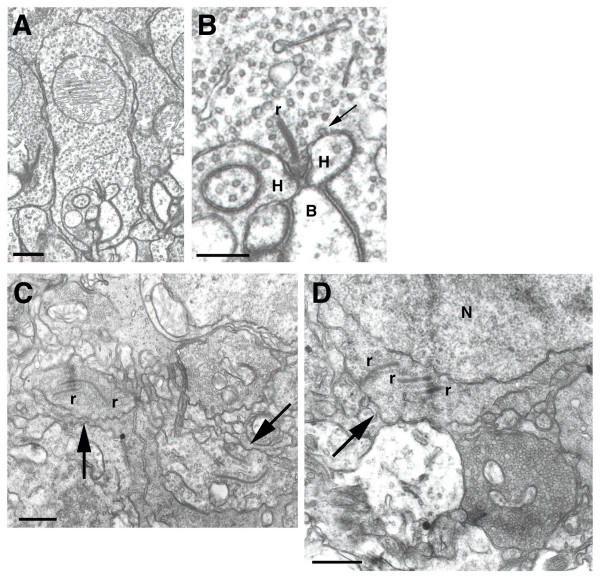
Transmission electron micrographs of the outer plexiform layer in Crx-/- retinas at P21. (A) Crx+/+ rod spherules contained a single invaginating synapse with one ribbon complex. The spherule was a blunt or club-shaped structure. (B) Crx+/+ rod terminals contained a single ribbon structure (r). Two processes, from horizontal cells (h), contacted the rod laterally. An additional process, the postsynaptic bipolar dendrite (b), was situated more centrally. Terminals were filled with synaptic vesicles. One coated vesicle originatinf from the cell membrane was observed (arrow). (C) In the OPL of Crx-/- retinas, photoreceptor terminals appeared highly disorganized. Putative terminals containing synaptic vesicles and ribbon-like structures were apparent (arrows), suggesting at least limited generation of synapse components. However, well formed spherules and pedicles were not observed. Further, many terminals appeared to contain multiple ribbon-like structures (r). The majority of these ribbons were not associated with the synaptic membrane, but instead were found free floating and, in some instances, were perinuclear (D, arrow). H, horizontal cell; B, bipolar cell; N, nucleus; r, ribbon. (A) Scale bar = 500 nm, (B) Scale bar = 250 nm, (C and D) Scale bar = 500 nm.

In a previous study, we demonstrated that forced expression of a dominant-negative allele of Crx in developing rods blocked formation of both rod spherules in the outer plexiform layer (OPL) and outer segments [7]. To expand on these studies, the ultrastructure of photoreceptor synapses was examined in Crx-/- retinas. In Crx+/+ retinas at P21, newly mature rod spherules were abundant (Figure 7A). The sperules were blunt or club-shaped structures with a single ribbon associated with a single invaginating synapse (Figure 7A, arrow indicates one example; Figure 8A and 8B). Two processes from horizontal cells were situated on either side of the synaptic ridge (Figure 8B, labelled H) and one or more dendrites of rod bipolar cells occupied a central position (Figure 8B, bipolar labelled B). Cone terminals are large, flat pedicles that exhibit many invaginating synapses containing separate sets of horizontal and bipolar cell processes. Each pedicle contains numerous ribbons. These terminals were much less common than spherules in Crx+/+ retinas at P21 (Figure 7, box). In the OPL of Crx-/- retinas, photoreceptor terminals were highly disorganized at P21 (Figure 7B, arrows). Processes containing synaptic vesicles and ribbon-like structures were apparent, suggesting at least limited generation of synapse components. However, well formed spherules and pedicles were not observed. In addition, many terminals appeared to contain multiple ribbons (Figure 8C and 8D, r) not tethered to the plasma membrane and occasionally in perinuclear positions (Figure 8D).

## Discussion

In this study, an ultrastructural analysis of Crx-/- photoreceptors was carried out. As Crx mutations have been associated with Leber's congenital amaurosis, the findings in this study broaden our understanding of the pathology of this disease. Two prominent pathologies were characterized in the Crx-/- retina: (1) An absolute block in outer segment morphogenesis was noted, with the block occuring at the elongation stage of outer segment formation; (2) Crx-/- photoreceptors exhibited a severe perturbation in synapse formation. This represents the first report of a synaptogenesis defect in an animal model of LCA.

### Crx-/- photoreceptors cannot complete outer segment morphogenesis

Mutations in Crx represent one of a collection of gene mutations that lead to an outer segment formation defect. Homozygous null mutations in the peripherin/RDS gene [36] or in rhodopsin [29] lead to a failure of outer segment formation. The deficits in peripherin-/- and Crx-/- photoreceptor morphogenesis were found to be very similar. Vesicular structures in Crx-/- photoreceptors were observed that were similar to those previously noted in the rds/peripherin-/- mouse. It was initially proposed that these vesicles were due to the breakdown of outer segment membranes that were not properly recruited to the outer segments in the absence of peripherin, or were from the result of the breakdown of the microvilli of Müller cells [30]. Strong support in favor of the former explanation was provided by Nir and colleagues who demonstrated the presence of rhodopsin protein in these vesicles using immunoelectron microscopy against a rhodopsin epitope [37]. Further, as shown here, the vesicles appear to bud from the inner segments themselves.

In developing photoreceptors, an extraordinary growth process occurs whereby the outer segment is generated from the nascent connecting cilium (see [38] and references therein). Peripherin/RDS and ROM-1 proteins (localized in disc rims) and the opsin proteins (localized throughout the discs) have important roles in the structural integrity of mature outer segments (see [39,29]). ROM-1-/- mice produce disorganized outer segments with large disks [40]. Crx, by virtue of being a transcription factor, presumably controls genes that are responsible for the building and perhaps maintenance of the outer segment structure, including rhodopsin and peripherin. Using northern blots [34], microarrays [10], and serial analysis of gene expression (SAGE) [35], we have defined a large number of genes that are altered in their expression level in Crx-/- mice. We found that rhodopsin expression was severely diminished in Crx-/- animals, and peripherin mRNA was reduced by approximately 30%. Transgenic mice with variable levels of expression of wild type rhodopsin exhibit rod degeneration [41], indicating the importance of the level of rhodopsin expression. In addition, the timing of rhodopsin expression may be very important, as indicated by studies in Drosophila.

In *Drosophila*, rhodopsin (*ninaE*) is expressed in photoreceptors R1–R6. In *ninaE *null mutants, the rhabdomere, a structure analogous to vertebrate outer segments, fails to develop in R1–R6 photoreceptors [42], reminiscent of the situation in rhodopsin-/- mice [29]. An intriguing experiment by Kumar et al. [43] demonstrated a temporal requirement for rhodopsin expression during rhabdomere development. In *ninaE *null flies, a *ninaE *transgene under the control of a heat shock promoter was subjected to various temperature shifts during development. Heat shock during the normal time of rhodopsin onset resulted in substantial and long-lasting rescue of photoreceptor structure and transient rescue of photoreceptor physiology. However, expression shortly before or after this critical period failed to rescue, suggesting that rhodopsin expression during a discrete window of time in development is essential for proper rhabdomere morphogenesis. This result is consistent with observations in the rat wherein rhodopsin onset occurs with strict timing in the developmental history of most rods in vivo [44]. Thus, through its regulation of rhodopsin levels, or perhaps through control of the kinetics of the up-regulation of rhodopsin beginning at about P6, Crx may be regulating outer segment morphogenesis. The similarty of the two cases may extend further. At present, the closest Crx relative in Drosophila is Otd, the founding member of the class of homeobox genes to which Crx belongs. Interestingly, in one hypomorphic allele of *Drosophila otd*, *otd*^*uvi*^, photoreceptor morphogenesis is also disrupted [45].

We found that there are many other genes that are dependent upon Crx. Those that are expressed at a lower level in the Crx-/- retina, such as rhodopsin and peripherin, comprise many that are either enriched or specific to photoreceptors in their expression [35]. They include enzymes that are important in lipid metabolism, protein folding and transport, as well as in other processes that one might envision would be important in building a structure such as the outer segment. In situ hybridization using probes from this collection of genes has revealed that some of them have their RNA localized to the inner segment, a finding typical for proteins targeted to the outer segment. Future analyses of the function of these genes might reveal their role in outer segment biogenesis.

Finally, polarization of photoreceptors was found to be largely intact, as was ciliogenesis. Another LCA gene, CRB1, and a related gene CRB3, have been implicated in ciliogenesis in *in vitro *models [46]. The *Drosophila *homologue of CRB1, Crumbs, has been implicated in photoreceptor morphogenesis [47]. However, the spontaneously occurring mouse mutant in CRB1, the Rd8 mouse, develops shortened outer segments that subsequently degenerate [48], suggesting that photoreceptor polarization and synaptogenesis are intact in this mutant. While CRB1 and Crx have been both linked to LCA, further work is necessary to determine if their function is linked in retinal development.

### Synaptogenesis is perturbed in Crx-/- photoreceptors

The Crx-/- mouse demonstrates the most severe abnormality of photoreceptor synapses reported to date. The peripherin-/- mouse develops a normal complement of photoreceptor terminals which then degenerate as the photoreceptors are lost [30]. Also, similarly in rhodopsin (Rho) and cyclic nucleotide-gated channel, alpha-3 (CNGA3) double knockout mice (Rho-/-, CNGA3-/-), synapses are reported to form normally [49]. These observations demonstrate that photoreceptor synaptogenesis can occur in the absence of outer segment formation. In keeping with this observation is the fact that some electroretinogram activity is present in peripherin-/- mice, suggesting that minimal phototransduction is present in these mice, enough to drive activity at the photoreceptor synapse. In vitro studies wherein synapse elements are formed in the absence of proper outer segment development and, therefore, possible absence of light-dependent photoreceptor activity, have indicated the independence of phototransduction and synapse formation, at least for the initial stages [50,51]. These data then suggest that the fact that the Crx-/- photoreceptors do not have proper synaptic endings is not due to a lack of outer segment formation. A more likely explanation is that Crx plays a role in photoreceptor synapse formation, perhaps by regulating directly, or indirectly, important genes in this process. Using immunohistochemistry, we examined the expression of common pre-synaptic terminal proteins, including KIF3a, SV2, and synaptophysin, and were unable to observe qualitative differences between Crx-/- and control tissue at P14 (data not shown). Examination of their RNA levels by SAGE showed no significant difference for all 3 genes, though very few tags were recovered from these genes and thus the analysis of RNA levels may not be significant [35]. However, since other genes expressed in photoreceptors were significantly altered in their expression level in the Crx-/- mouse, there are many candidates that could be important for photoreceptor morphogenesis. Tags from three genes from proteins expressed in photoreceptor terminals were found to be decreased in a statistically significant fashion, namely the HGF-regulated tyrosine kinase substrate, the CRIPT protein, and synaptotagmin 1 (Blackshaw and Cepko, unpublished data). An example of a gene that was increased in the Crx-/- retina is HRG4 (a homologue of the C. elegans Unc119 gene) (Blackshaw and Cepko, unpublished data) which encodes a component of the ribbon synapse [33]. The fact that it is upregulated might indicate a response to the lack of proper terminal structures. Several other genes encoding putative cytoskeletal elements also were increased (e.g. microtubule associated protein 4) or decreased (e.g. cofilin 1) in the Crx-/- retina, with P values of <.005. It is not known whether any of these genes are involved in building or regulating synaptic structures, but they are now genes that might lead to a better understanding of the construction and function of the relatively unique structure of the ribbon synapse.

Abnormal photoreceptor terminal formation was noted in a study that examined retinal development in the laminin beta2 chain-deficient mouse [52]. Several pathologies were noted in these mice. First, laminin beta2 chain-deficient mice displayed abnormal outer segment elongation, but a more mild phenotype than that of the Crx-/- mice; the outer segments were reduced by 50% in length. Also photoreceptor terminals were perturbed in laminin beta2 mutants, but again the phenotype was more subtle then that of Crx-/- mice. The outer plexiform layer of the beta2-deficient retinas demonstrated only 7% normal invaginating synapses, while the remainder had various pathologies, including floating synaptic ribbons, as seen here. The mechanistic relationship of these two molecules, if any, in photoreceptor morphogenesis is unknown to date. The mRNA for laminin beta2 was not detected in the SAGE study of the relative RNA levels in Crx-/- and Crx+/+ and thus we cannot comment on whether the levels of RNA for laminin beta2 were altered.

### Crx-/- mice are a model for LCA

Crx has been implicated in three photoreceptor diseases that result in human blindness, cone-rod dystrophy2, Leber's congenital amaurosis, and retinitis pigmentosa (for review, see [53]). The cone-rod dystrophies (CRDs) are characterized by loss of cone-mediated vision in the first decade of life or later, with concomitant or subsequent loss of rod-mediated vision [54]. Conversely, RP is notable for initial loss of rod function, followed by loss of cone-mediated vision [55]. The majority of known genes responsible for human genetic blindness, encode proteins expressed almost exclusively, or exclusively, in photoreceptors, particularly in the outer segment [35]. Many of these proteins are required for phototransduction or outer segment structure. The mechanisms whereby mutations in rod-specific genes, such as rhodopsin, lead eventually to cone degeneration in RP remain obscure. Mutations in Crx were the first, and still one of a very few examples of a transcription factor mutation leading to photoreceptor disease.

LCA is a disease in which there is little or no photoreceptor function in infancy; thereby, likely developmental in etiology ([17,56] for review). The Crx-/- mouse may be an excellent model for studying the pathology of this disorder, particularly the subtype of the disorder where Crx mutations are involved. The vast majority of histopathological studies of LCA in human tissue have been derived from adult patients with LCA where secondary changes are likely to be present. Indeed in animal models of LCA, secondary reactive and/or degenerative changes occur early after the abnormal formation of retinal tissue [57]. The only study in human tissue derived from a human 33-week retina with proposed RPE65 mutations was reported to have abnormal retinae at this early stage [24]. These authors report cell loss, including thinning of the photoreceptor layer. In addition, they claim in the text to have seen aberrant synaptic and inner retinal organization, although their examination of photoreceptor synapses unfortunately are not presented in the data section of the paper. Given the scarcity of available human tissue, the characterization of the primary pathology of LCA will require animal models. In the current study, we present data that argue that, in addition to outer segment morphogenesis, synaptogenesis may also be critically impaired in at least a subset of LCA.

## Methods

### Mice

Crx-/- mice were generated as reported elsewhere [34]. Rhodosin-null mice [29] were obtained from Paul Sieving (University of Michigan). Rds mice were acquired from Jackson Laboratory.

### Transmission electron microscopy

Littermate Crx-/- and wildtype pups were perfused in 1% formaldehyde and 0.5% glutaraldehyde at various postnatal stages. The eyes were then enucleated, and the cornea and lens were removed. The eye cup was immersed in fixative (1% formaldehyde and 2.5% glutaraldehyde) for 3 to 4 hours at 4°C. The sclera was then partially removed and the retinas were sliced into small pieces and fixed (1% paraformaldehyde and 2.5% glutaraldehyde) overnight at 4°C. These procedures were found optimal for maintaining the structural integrity of the photoreceptor outer segments.

After fixing, the tissue was washed 2X in PBS for thirty minutes per wash. The tissue was then postfixed in a 1% osmium tetroxide/1.5% potassium ferrocyanide mixture for 2 hours at 4°C. Staining was carried out for 30 minutes in 1% uranyl acetate in maleate buffer (pH = 6.0) at room temperature followed by 1% tannic acid in 0.1 M cacodylate buffer (pH = 7.4) for thirty minutes. The specimens were then dehydrated and embedded in Epon/Araldite. Thin sections were stained with uranyl acetate and lead citrate, and examined in a Jeol JEM-1200EX electron microscope.

### Scanning electron microscopy

Specimens used for SEM required removal of the retinal pigment epithelium (PE), enabling visualization of the outer surface of the neural retina. Retinae from Crx-/-, rhodopsin-/-, RDS, or wildtype eyes were dissected free from PE in a dispase solution and fixed in 1.25% glutaraldehyde and 1.0% formaldehyde overnight at 4°C. Tissue was then washed 5X in cacodylate buffer and dehydrated in ascending grades of ethanol. Tissues were subsequently critical point dried in carbon dioxide. All specimens were mounted and coated with sublimated gold-palladium by the sputtering technique. Micrographs were obtained with a Jeol JSM-35CF scanning electron microscope.

## Authors' contributions

EM and ER conducted transmission electron microscopy. EM performed scanning electron microscopy. TF and EM generated, characterized and maintained the Crx-/- mouse line. CC participated in the design and coordination of the study and all data analysis. EM and CC drafted the manuscript. All authors read and approved the final manuscript.
